# *CERKL-*related inherited retinal dystrophy in a Brazilian cohort: genotype-phenotype correlation

**DOI:** 10.3389/ebm.2026.10935

**Published:** 2026-09-23

**Authors:** E. S. Yasaki, M. V. Salles, F. L. Motta, J. M. F. Sallum

**Affiliations:** 1 Department of Ophthalmology and Visual Sciences, Universidade Federal de São Paulo, São Paulo, Brazil; 2 Instituto de Genética Ocular, São Paulo, Brazil

**Keywords:** *CERKL* gene, cone rod dystrophy, inherited retinal dystrophy, macular dystrophy, retinitis pigmentosa

## Abstract

The purpose of this study was to analyze the genotype-phenotype of *CERKL*-related inherited retinal dystrophy in an outpatient clinic. For the study, 2841 medical records of Brazilian patients with a diagnosis of an inherited retinal dystrophy (IRD) registered at Instituto de Genética Ocular, Brazil, between January 2006 and July 2025 were retrospectively reviewed and 52 patients from 50 unrelated families with a molecular diagnosis of an IRD related to the *CERKL* gene were selected. Clinical data and molecular tests results were analyzed for genotype-phenotype correlation. Most patients (50/52) presented with the cone rod dystrophy (CORD) and two patients presented with the macular dystrophy (MD) phenotype. Age of presenting symptoms ranged from early childhood (7 years old) to adulthood (40 years old). BCVA Snellen measured at the first visit ranged from 20/25 to light perception, corresponding to 0.1 to 2.7 in log MAR visual acuity. Visual acuity data according to disease duration at the first visit of 37 double null (DN) alleles patients disclosed a Pearson coefficient R^2^ of 0.315 for the right eye and 0.405 for the left eye, respectively. A highly significant association between disease duration and visual acuity loss was found suggesting consistent and predictable visual deterioration in patients with complete CERKL loss-of-function over time. Twenty-four different variants described in the *CERKL* gene were identified. The c.847C>T (p.Arg283) nonsense variant in exon 6 was the most common variant in this cohort, identified in 24 homozygous out of 52 individuals and in 12 compound heterozygous. The phenotype of *CERKL*-related IRD is CORD with an early age of presentation and fast decrease in visual acuity. In addition to OMIM classifying this gene as an retinitis pigmentosa IRD, we believe *CERKL* has CORD phenotype, in concordance with evidence described in the literature.

## Impact statement

The work intersects clinical genetics, retinal dystrophy and variant interpretation, delivering guidance for clinical reporting and strengthening the evidence that *CERKL* is also a contributor to inherited retina dystrophy. To date, data on the prevalence of autosomal-recessive retinal dystrophies in the Brazilian population are scarce; specifically, for *CERKL*, this is the first report to describe a genotype-phenotype correlation. This paper may contribute to increase the knowledge of populational data and understanding the genetic bases in a diverse ethnic population. We believe this will interest clinicians and researchers working on retina degeneration and gene-level mechanisms.

## Introduction

Inherited retinal dystrophies (IRD) are a heterogenous group of ocular diseases characterized by progressive photoreceptor degeneration due to apoptosis, leading to irreversible blindness. Retinitis pigmentosa (RP; MIM 268000) is the most common IRD; more than ninety genes have been implicated in the autosomal-recessive form to date [1]. Cone-rod dystrophy (CORD) is a rarer IRD, with an estimated prevalence of approximately 1:40,000 in Europe [2]. Unlike typical RP, CORD is characterized by primary cone dysfunction followed by secondary rod degeneration [3]. Classical clinical features include progressive loss of central vision, color-vision disturbance, and photophobia, followed by nyctalopia and peripheral field constriction. Overall, CORD tends to progress more rapidly and patients reach legal blindness earlier than those with retinitis pigmentosa.

*CERKL*-related retinopathy (MIM 608380), recorded as RP26 in OMIM, accounts for an estimated 4–7% of autosomal-recessive IRD cases across diverse populations [4–6]. In founder populations, the proportion can be substantially higher, up to 33% in specific cohorts due to founder effects [7–11].

Ceramide kinase-like (*CERKL*) gene has been first mapped in chromosome 2q31.2-q32 in a consanguineous Spanish family with autosomal recessive RP (arRP) in 1998 [12]. A few years later, Tuson and colleagues identified a homozygous mutation in exon 5 of CERKL in this same Spanish family and in an additional unrelated family [7]. This report was the first to provide evidence suggesting a link between retinal degeneration in RP and sphingolipid-mediated apoptosis. The *CERKL* gene shows higher transcription levels in the retina and is also expressed in other tissues, including the brain, lung, and kidney [13].

To date, data on the prevalence of autosomal-recessive retinal dystrophies in the Brazilian population are scarce; specifically, for *CERKL*, this is the first report to describe a genotype–phenotype correlation.

Using the transcript NM_001030311 as the reference, *CERKL* comprises 14 exons and encodes a 558 amino acid protein. This isoform comprises a diacylglycerol kinase domain (DAGK) and a putative N-terminal pleckstrin homology (PH) region.^13^ Different retinal isoforms produce multiple transcripts as a result of alternative splicing [14].

*CERKL* genes show 29% identity and 50% similarity with the human ceramide kinase (CERK) protein [15] which participates in oxidative stress protection, but no kinase activity was shown [16]. The exact role of CERKL remains unclear. A recent study using a CERKL knockdown mouse model, generated by CRISPR-based gene silencing, demonstrated an inability to activate cell protection upon light stress conditions [17]. *CERKL* is a resilience gene in oxidative stress; therefore, altered expression may trigger retinal degeneration through apoptosis and non-apoptotic inflammatory response mediated by glia.

Ceramide is a sphingolipid that has a central role in lipid metabolism, both structural for the lipid cell membrane composition and metabolic. Ceramide acts as a signaling lipid in multiple cellular pathways that regulate cell growth, cell differentiation, senescence, and apoptosis. Ceramide level increase or imbalance triggers photoreceptor degeneration, induces amacrine cell death, and contributes to retinal pigmented epithelial (RPE) cell dysfunction and atrophy.

Sphingosine (Sph) is an 18-carbon, long chain amino alcohol, the canonical sphingoid base d18:1, that serves as the common backbone of most sphingolipids. The ceramide structure is formed by n-acylation of sphingosine with fatty acid, creating an amid bond, N-acylsphingosine. Figure 1 displays how sphingolipid intermediate metabolites and ceramides are interconnected in a network.

**FIGURE 1:**
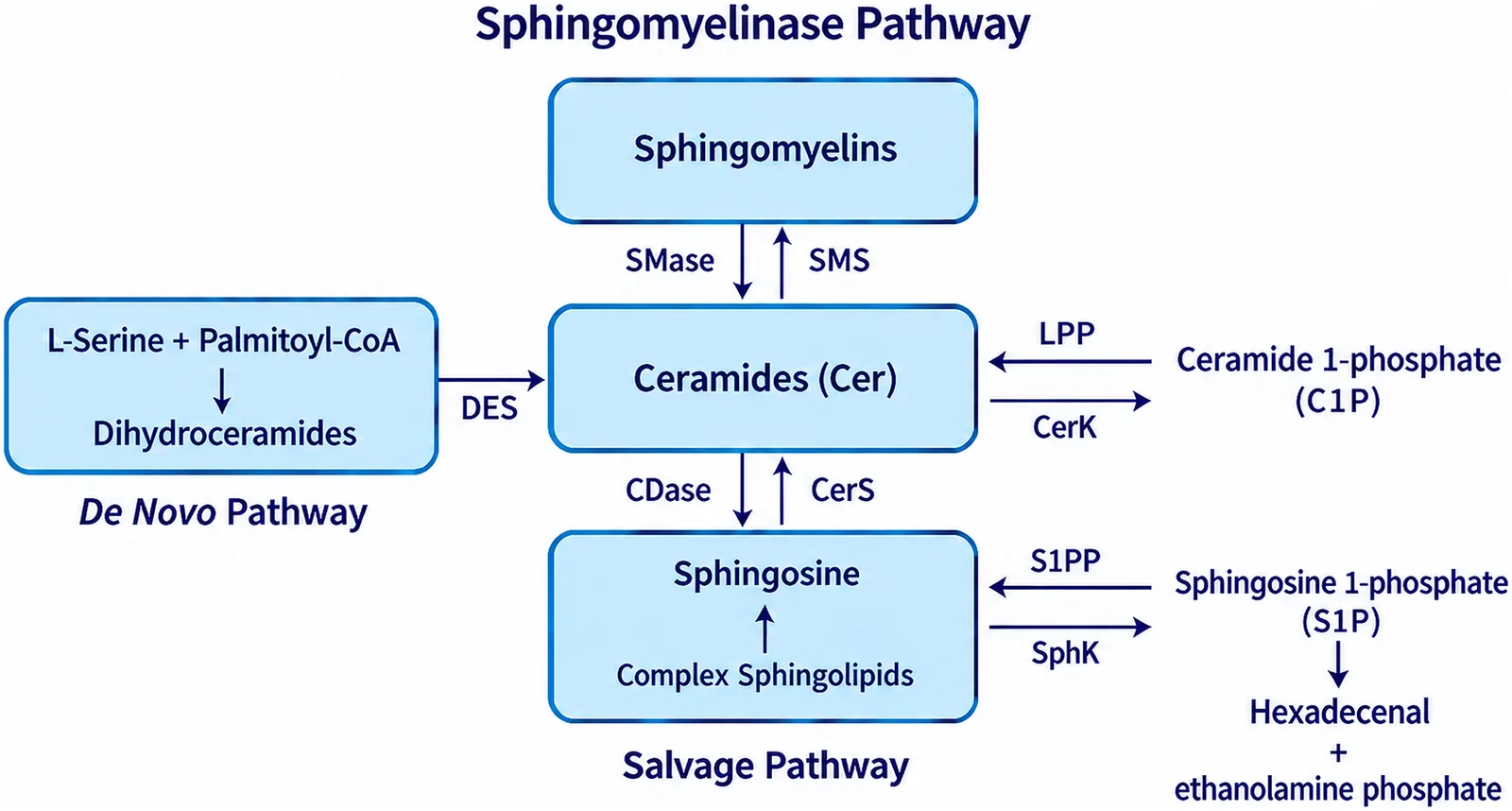
Sphingolipid and ceramide network.

## Materials and methods

The clinical notes of 2841 Brazilian patients diagnosed with IRD registered at Instituto de Genética Ocular between January 2006 and July 2025 were reviewed, from which fifty-seven patients with genetic testing suggestive of *CERKL*-related inherited retinal disease (IRD) were identified. Four patients were excluded due to missing clinical information. One patient, listed as 45.1 and identified as homozygous for a variant of uncertain significance (VUS), was excluded from the analysis. Data were obtained from clinicians’ notes detailing ophthalmological exams performed by specialists in IRD. Molecular test results were collected and analyzed for genotype-phenotype correlation. Ethical approval was provided by the Research Ethics Committee of Federal University of São Paulo (number 1191/2018) and the study complied with the tenets of the Declaration of Helsinki. When the DNA samples were collected for molecular tests, all patients and/or their legal guardians provided written informed consent for the use of their personal medical data for scientific purposes and publication.

Clinical data included detailed ocular examination findings, presenting signs and symptoms, age at disease onset, Snellen best corrected visual acuity (BCVA) measured at first visit, which was converted to log Mar [18, 19], slit-lamp biomicroscopy, fundus examination, digital color fundus picture, and, if available, fundus autofluorescence, spectral domain optical coherence tomography, and electrophysiological test. Phenotype classification was based primarily on clinical data and fundus picture analysis performed by specialists in IRD.

*CERKL* variants were identified by the panel sequencing of genes associated with inherited retinal disease performed in different laboratories using panel-based targeted next-generation sequencing (NGS) or whole-exome sequencing. The pathogenicity of variants was determined by the American College of Medical Genetics (ACMG) and the Association for Molecular Pathology (AMP) criteria [20]. Novel variants were classified as pathogenic or likely pathogenic according to the criteria of effect in the protein structure (frameshift or nonsense of copy number variation or affecting a canonical splice site), frequency in the gnomAD population database, and classification by pathogenic predictors. Two platforms were assessed to assist variant interpretation, namely Franklin (https://franklin.genoox.com) and Varsome (https://varsome.com), assessed on 4 Sep 2025. Variants found in the cohort were compared with the listed ones in Clin Var Nucleotide (https://www.ncbi.nlm.nih.gov/clinvar/assessed on 4 Sep 2025), and protein numbering is based on *CERKL* transcript NM_01030311 (Figure 2). Owing to the retrospective design of the study and the lack of available parental specimens, segregation analysis was not performed for any of the families in this cohort.

**FIGURE 2:**
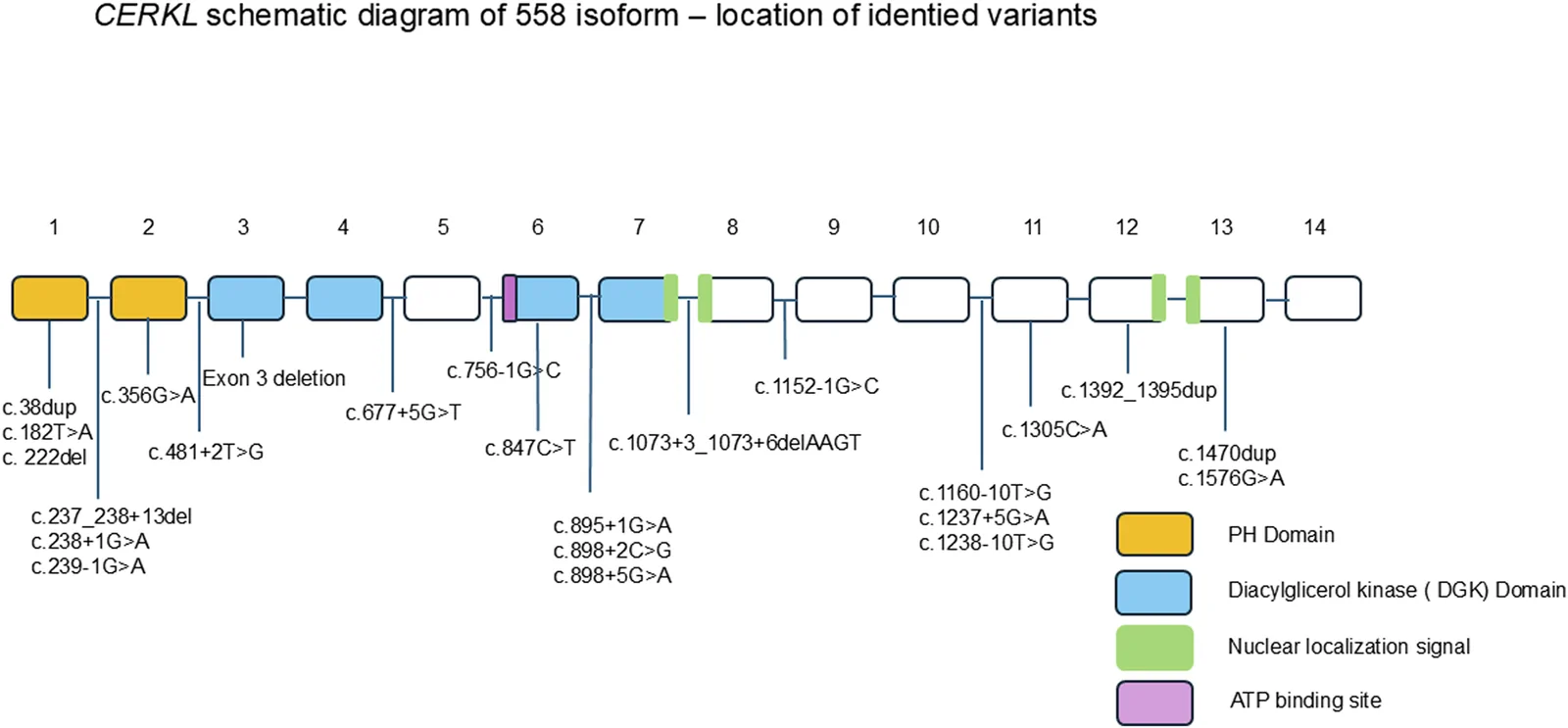
*CERKL* schematic diagram of 558 isoforms and location of identified mutations.

## Results

### Demographics

The study included fifty-two individuals of 50 pedigrees, and their clinical characteristics, variant description, and zygosis are described in Table 1. Twenty-two patients were female and thirty were male. The median age at presentation was 18 years, ranging from early childhood (7 years old) to adulthood (40 years old). Consanguinity was reported in fifteen families (15/50). Figure 3 show fundus aspect of the homozygous c.847C>T variant ranging from subtle to severe atrophy and visual impairment. Figure 4 show fundus features of the homozygous variants.

**TABLE 1:** Clinical features, description of *CERKL* variants, and zygosis.

#Family	#Patient	Age at onset	Age of 1st visit (years)	Clinical diagnosis/	Signs/ Symptoms	Vision acuity	DNA variant	Protein change	Zygosity	Variant classification (ACMG)
		(years)		Phenotype		(logMAR)				
F1	1.1	14	45	Cone rod	Poor visual acuity/Nyctalopia/ VF constriction	2.3	c.847C>T	p.Arg283*	HOMO	Pathogenic
						2.3				
F2	2.1	19	36	Cone rod	Poor visual acuity/ VF constriction	0.7	c.356G>A	p.Gly119Asp	HOMO	Pathogenic /Likely Pathogenic
						1.3				
F2	2.2	18	24	Cone rod	Poor central visual acuity/Photophobia	0.1	c.356G>A	p.Gly119Asp	HOMO	Pathogenic/Likely Pathogenic
						0.4				
F3	3.1	12	29	Cone rod	Poor central visual acuity /Nyctalopia	1.9	c.847C>T	p.Arg283*	HOMO	Pathogenic
						1.9				
F4	4.1	16	24	Cone rod	Poor visual acuity	0.6	c.222del	p.Gln74Hisfs*28	HETERO	Pathogenic
						1.6	c.847C>T	p.Arg283*	HETERO	Pathogenic
F5	5.1	30	35	Cone rod	Poor visual acuity	n/a	c.847C>T	p.Arg283*	HOMO	Pathogenic
F6	6.1	14	19	Cone rod	Poor visual acuity /Nyctalopia	0.5	c.847C>T	p.Arg283*	HOMO	Pathogenic
						0.5				
F7	7.1	16	44	Cone rod	Poor visual acuity/ VF constriction	2.7	c.847C>T	p.Arg283*	HOMO	Pathogenic
						2.7				
F8	8.1	17	28	Cone rod	Poor visual central visual acuity/ Color vision disturbance /nyctalopia / VF constriction	n/a	c.237_238+13del	p.Gly80fs	HOMO	Pathogenic
F9	9.1	24	26	Cone rod	Poor central visual acuity	0.9	c.1305C>A	p.Cys435*	HOMO	Pathogenic
						0.9				
F10	10.1	18	31	Cone rod	Poor visual acuity/Photophobia / VF constriction	0.6	c.847C>T	p.Arg283*	HOMO	Pathogenic
						1.9				
F11	11.1	15	20	Cone rod	Poor visual acuity/Nyctalopia / Poor color discrimination	0.4	c.847C>T	p.Arg283*	HETERO	Pathogenic
						0.4	c.356G>A	p.Gly119Asp	HETERO	Likely pathogenic /Pathogenic
F12	12.1	21	26	Cone rod	Poor central visual acuity	1.9	c.237_238+13del	p.Gly80fs	HOMO	Pathogenic
						1.9				
F13	13.1	16	27	Cone rod	Poor central visual acuity/Nyctalopia	0.9	c.847C>T	p.Arg283*	HOMO	Pathogenic
						0.7				
F14	14.1	21	43	Cone rod	Poor central visual acuity/Nyctalopia/Photophobia	2.0	c.239-1G>A	p.?	HOMO	Pathogenic
						2.0				
F15	15.1	20	50	Cone rod	Poor central visual acuity/Photophobia	n/a	c.898+2C>G	p.?	HOMO	Pathogenic
F16	16.1	21	23	Cone rod	Poor central visual acuity	0.5	c.847C>T	p.Arg283*	HETERO	Pathogenic
						0.5	c.1238-10T>G	p.?	HETERO	Likely pathogenic
F17	17.1	13	32	Cone rod	Nyctalopia/poor visual acuity/photophobia/poor color discrimination	0.7	c.847C>T	p.Arg283*	HETERO	Pathogenic
						0.6	c.356G>A	p.Gly119Asp	HETERO	Likely pathogenic/Pathogenic
F18	18.1	38	43	Cone rod	Poor visual acuity/Nyctalopia/Photophobia/VF constriction	0.3	c.847C>T	p.Arg283*	HOMO	Pathogenic
						0.5				
F19	19.1	19	40	Cone rod	Poor central visual acuity/Photophobia/nyctalopia/poor color discrimination	2.7	c.847C>T	p.Arg283*	HOMO	Pathogenic
						2.7				
F20	20.1	24	44	Cone rod	Poor visual acuity/Nyctalopia/VF constriction	0.6	c.1238-10T>G	p.?	HETERO	Likely pathogenic
						0.6	c.895+1G>A	p.?	HETERO	Pathogenic
F21	21.1	19	28	Cone rod	Poor visual acuity/Nyctalopia/VF constriction	2.3	c.182T>A	p.Val61Glu	HOMO	Likely pathogenic
						0.3				
F22	22.1	7	24	Cone rod	Poor visual acuity/Nyctalopia	1.9	c.847C>T	p.Arg283*	HOMO	Pathogenic
						1.9				
F23	23.1	15	23	Cone rod	Poor visual acuity/Photophobia	0.7	c.847C>T	p.Arg283*	HETERO	Pathogenic
						0.6	c.1073+3_1073+6delAAGT	p.?	HETERO	Likely pathogenic
F24	24.1	12	37	Macular dystrophy	Poor central visual acuity	1.0	c.1576G>A	p.Asp526Asn	HETERO	VUS
						1.0	c.237_238+13del	p.Gly80fs	HETERO	Pathogenic
F25	25.1	27	27	Cone rod	Photophobia/Poor color discrimination	0.1	c.847C>T	p.Arg283*	HOMO	Pathogenic
						0.1				
F26	26.1	16	27	Cone rod	Poor visual acuity/Nyctalopia	0.6	c.847C>T	p.Arg283*	HOMO	Pathogenic
						1				
F27	27.1	14	38	Cone rod	Poor central and peripheral visual acuity	2.3	c.847C>T	p.Arg283*	HETERO	Pathogenic
						2.3	c.1470dup	p.Thr491Hisfs*3	HETERO	Pathogenic
F28	28.1	20	24	Cone rod	Poor visual acuity	n/a	c.847C>T	p.Arg283*	HOMO	Pathogenic
F29	29.1	23	30	Cone rod	Poor visual acuity/Nyctalopia/VF constriction	1.9	c.847C>T	p.Arg283*	HOMO	Pathogenic
						0.5				
F30	30.1	13	36	Cone rod	Poor visual acuity/Photophobia/Nyctalopia/VF constriction	0.5	c.847C>T	p.Arg283*	HOMO	Pathogenic
						0.5				
F31	31.1	12	38	Macular dystrophy	Poor visual acuity/Photophobia	1.9	c.1238-10T>G	p.?	HETERO	Likely pathogenic
						1.9	Deletion (exon 3)	p.?	HETERO	VUS
F32	32.1	13	17	Cone rod	Poor visual acuity/Photophobia	0.7	c.847C>T	p.Arg283*	HOMO	Pathogenic
						0.9				
F33	33.1	11	13	Cone rod	Poor central visual acuity/Nyctalopia/VF constriction/Color vision discrimination	0.4	c.847C>T	p.Arg283*	HOMO	Pathogenic
						0.5				
F34	34.1	21	26	Cone rod	Poor visual acuity/Nyctalopia	0.6	c.847C>T	p.Arg283*	HOMO	Pathogenic
						0.6				
F35	35.1	33	45	Cone rod	Poor visual acuity/Photophobia	n/a	c.237_238+13del	p.Gly80fs	HETERO	Pathogenic
							c.1237+5G>A	p.?	HETERO	VUS
F35	35.2	25	56	Cone rod	Poor visual acuity/Photophobia	n/a	c.237_238+13del c.1237+5G>A	p.Gly80fs p.?	HETERO HETERO	Pathogenic VUS
F36	36.1	15	17	Cone rod	Poor visual acuity/Color abnormalities/VF constriction	0.3	c.1392_1395dup	p.Thr465Leufs*4 p.?	HETERO	Pathogenic
						0.4	c.481+2T>G		HETERO	Pathogenic
F37	37.1	15	33	Cone rod	Poor visual acuity/VF constriction	2.7	c.847C>T	p.Arg283*	HOMO	Pathogenic
						2.7				
F38	38.1	12	42	Cone rod	Poor visual acuity	1.3	c.847C>T	p.Arg283*	HOMO	Pathogenic
						2.0				
F39	39.1	15	32	Cone rod	Poor visual acuity/Photophobia	2.0	c.239-1G>A	p.?	HETERO	Pathogenic
						2.0	c.847C>T	p.Arg283*	HETERO	Pathogenic
F39	39.2	13	30	Cone rod	Poor central visual acuity/Nyctalopia	0.6	c.239-1G>A	p.?	HETERO	Pathogenic
						0.5	c.847C>T	p.Arg283*	HETERO	Pathogenic
F40	40.1	13	24	Cone rod	Poor central visual acuity/Photophobia	2.2	c.239-1G>A	p.?	HETERO	Pathogenic
						2.2	c.847C>T	p.Arg283*	HETERO	Pathogenic
F41	41.1	n/a	19	Cone rod	Poor visual acuity	n/a	c.847C>T	p.Arg283*	HOMO	Pathogenic
F42	42.1	40	42	Cone rod	Poor visual acuity/Photophobia/Amblyopia OS	0.2	c.847C>T c.1152- 1G>C	p.Arg283*	HETERO	Pathogenic
						1.0		p.?	HETERO	Pathogenic
F43	43.1	21	52	Cone rod	Poor visual acuity	0.9	c.847C>T	p.Arg283*	HETERO	Pathogenic
						1.6	c.1238-10T>G	p.?	HETERO	Likely pathogenic
F44	44.1	14	32	Cone rod	Poor visual acuity/Photophobia/Nyctalopia/VF constriction	0.7	c.847C>T	p.Arg283*	HETERO	Pathogenic
						2.0	c.677+5G>T	p.?	HETERO	VUS
F45	45.1*	15	45	Cone rod	Poor visual acuity/Photophobia	2.7	c.898+5G>A	p.?	HOMO	VUS
						3.0				
F46	46.1	20	27	Cone rod	Poor visual acuity/VF constriction	1.3 1.0	c.847C>T	p.Arg283*	HOMO	Pathogenic
F47	47.1	40	62	Cone rod	Poor visual acuity	1.0	c.847C>T	p.Arg283*	HOMO	Pathogenic
						0.7				
F48	48.1	24	40	Cone rod	Poor visual acuity/Photophobia/Poor color discrimination/nyctalopia/VF constriction	n/a	c.847C>T	p.Arg283*	HOMO	Pathogenic
F49	49.1	35	59	Cone rod	Poor visual acuity/VF constriction	2.3	c.182T>A	p.Val61Glu	HOMO	Likely pathogenic
						2.3				
F50	50.1	27	29	Cone rod	Poor visual acuity	0.6	c.238+1G>A	p.?	HOMO	Pathogenic
						0.5				

* Patient 45.1 is a homozygous VUS, and was excluded from the analysis.

**FIGURE 3:**
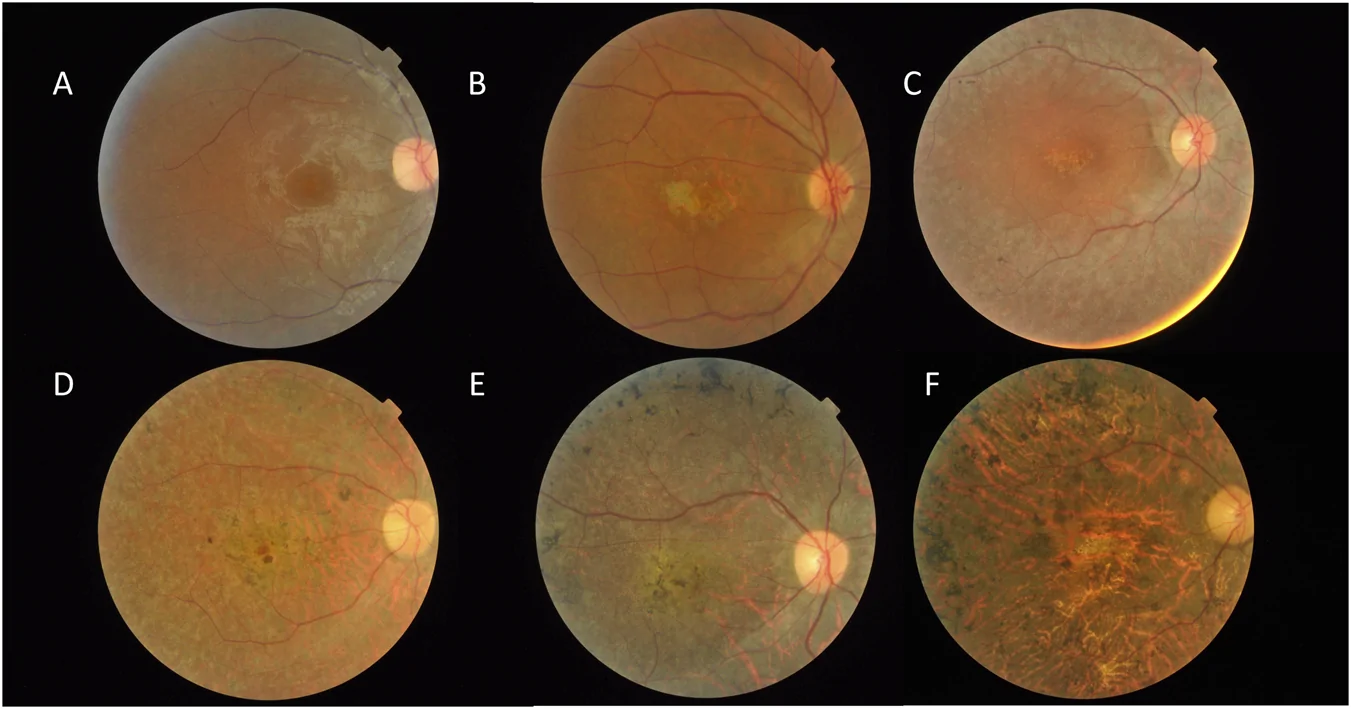
Fundus features of Homozygous c.847C>T variant **(A)** Patient 6.1, **(B)** Patient 29.1, **(C)** Patient 3.1, **(D)** Patient 11.1, **(E)** Patient 37.1, and **(F)** Patient 7.1.

**FIGURE 4:**
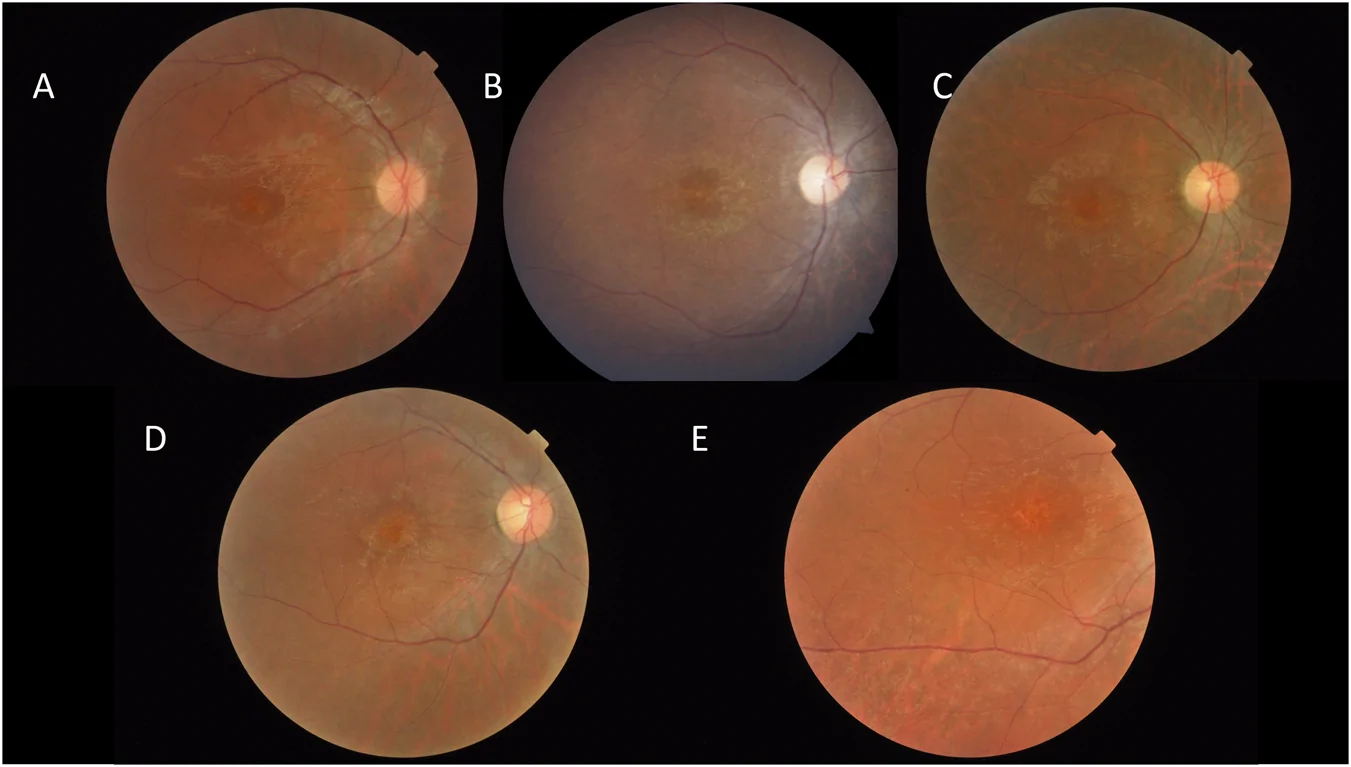
Fundus features of Homozygous variants **(A)** Patient 33.1 c.847C> T, **(B)** Patient 8.1 c.237_238_13del, **(C)** Patient 50.1 238+1G>A, **(D)** Patient 2.2 356G>A, and **(E)** Patient 9.1 1305C>A.

### Phenotype and visual acuity

The clinical diagnosis of all patients was reevaluated and reclassified. We concluded that all (50/52) but two patients exhibited a CORD phenotype. Macular dystrophy (MD) was the suggested phenotype for these two patients (2/52). Snellen BCVA, measured in the first visit, ranged from 20/25 to light perception, corresponding to 0.1 to 2.7 in log Mar visual acuity. Eight patients had no documented visual acuity. Figures 5 and 6 illustrate the data of visual acuity according to disease duration at the first visit of 37 double null (DN) alleles patients (Figure 5 for right and Figure 6 for left eye, respectively). Figure 7 and 8 illustrate the data of visual acuity according to disease duration at the first visit of 7 non-double null (NDN) patients (Figure 7 for right eye and Figure 8 for left eye, respectively). In the DN group (n = 37), a highly significant association between disease duration and VA loss in both eyes was found (RE: R^2^ = 0.315, β = 0.047 log MAR/year, p < 0.001; LE: R^2^ = 0.405, β = 0.053 log MAR/year, p < 0.001), suggesting a consistent and predictable visual deterioration in patients with complete *CERKL* loss-of-function over time.

**FIGURE 5:**
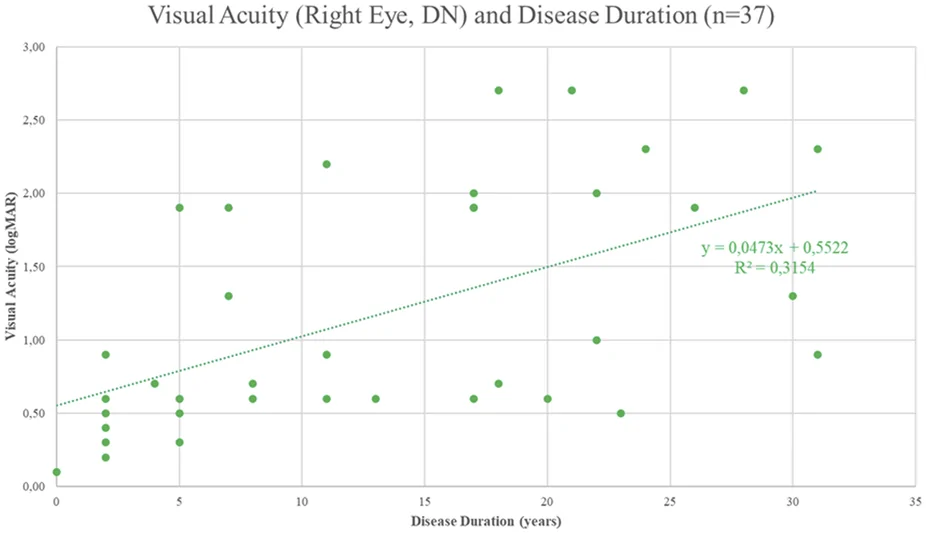
Visual acuity data according to disease duration at first visit of thirty-seven double null (DN) alleles patients for the right eye.

**FIGURE 6:**
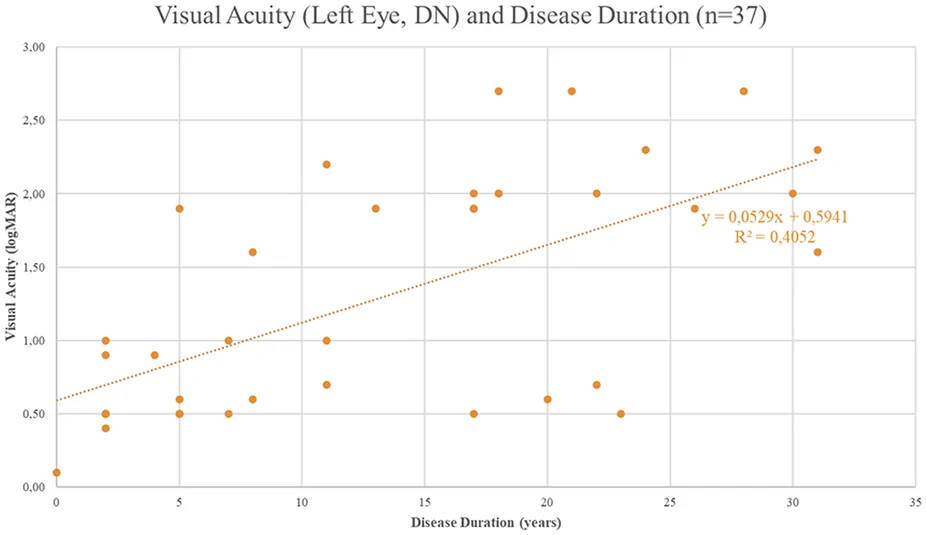
Visual acuity data according to disease duration at first visit of thirty-seven double null (DN) alleles patients for the left eye.

**FIGURE 7:**
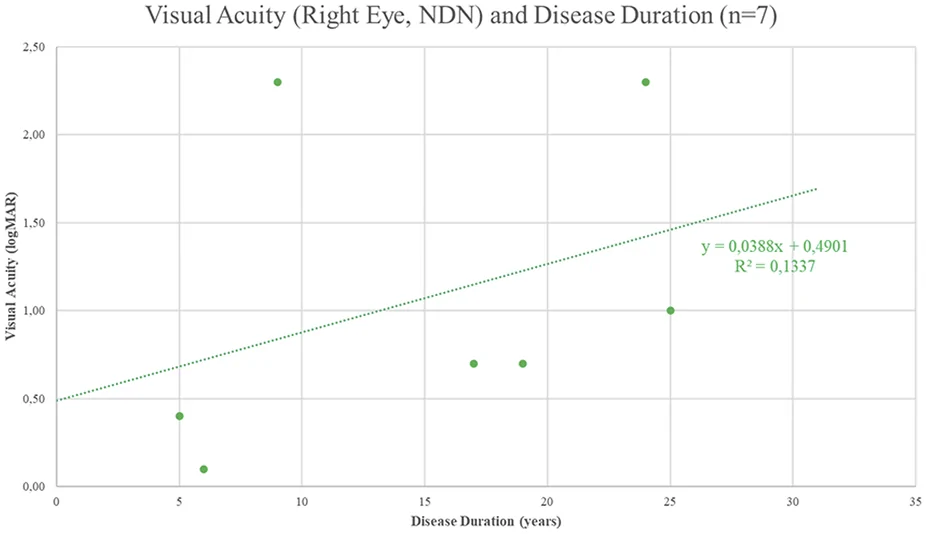
Visual acuity data according to disease duration at first visit of seven non-double null (NDN) alleles patients for the right eye.

**FIGURE 8:**
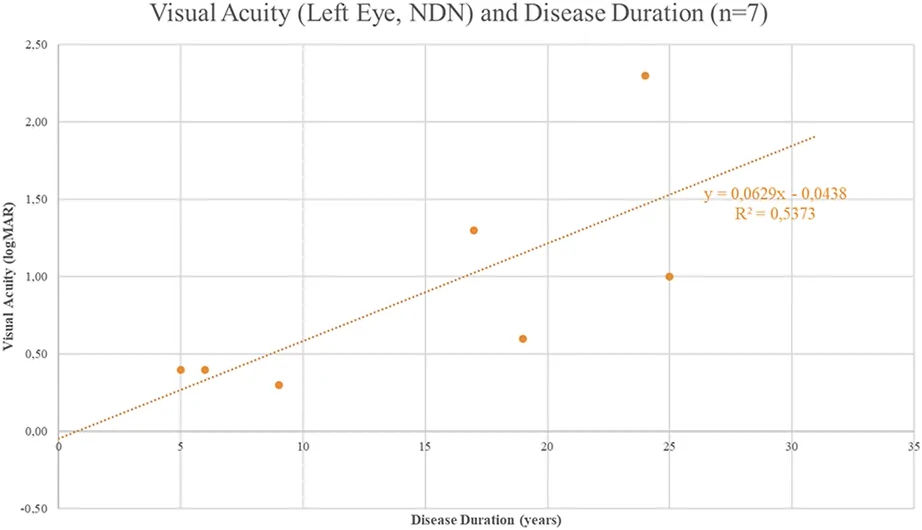
Visual acuity data according to disease duration at first visit of seven non-double null (NDN) alleles patients for the left eye.

On the other hand, the NDN group (n = 7) showed no statistically significant association between disease duration and VA in either eye (RE: R^2^ = 0.134, p = 0.420; LE: R^2^ = 0.537, p = 0.061). The small sample size of the NDN group (n = 7) limits statistical power and precludes definitive conclusions.

Age of onset, signs, and symptoms of all patients are listed in Table 1.

### Clinical examination, color, and fundus autofluorescence imaging

Retinography was available for 46 patients; fundus autofluorescence was available for 2. All available images (46/46) showed early macular involvement. Eleven patients (11/46) had no pigment deposits, twenty (20/46) had minimal or sparse pigmentation, and sixteen (16/46) had diffuse and dense bone-spicule-like pigmentation. Electroretinography (full field ERG) findings were available in three homozygous c.847C>T individuals (3/52), two of them (patients 6.1 and 13.1) with a full field extinct pattern and one with abnormal cone response and extinct rod response (patient 18.1). Figure 9 shows Patient11.1 fundus features.

**FIGURE 9:**
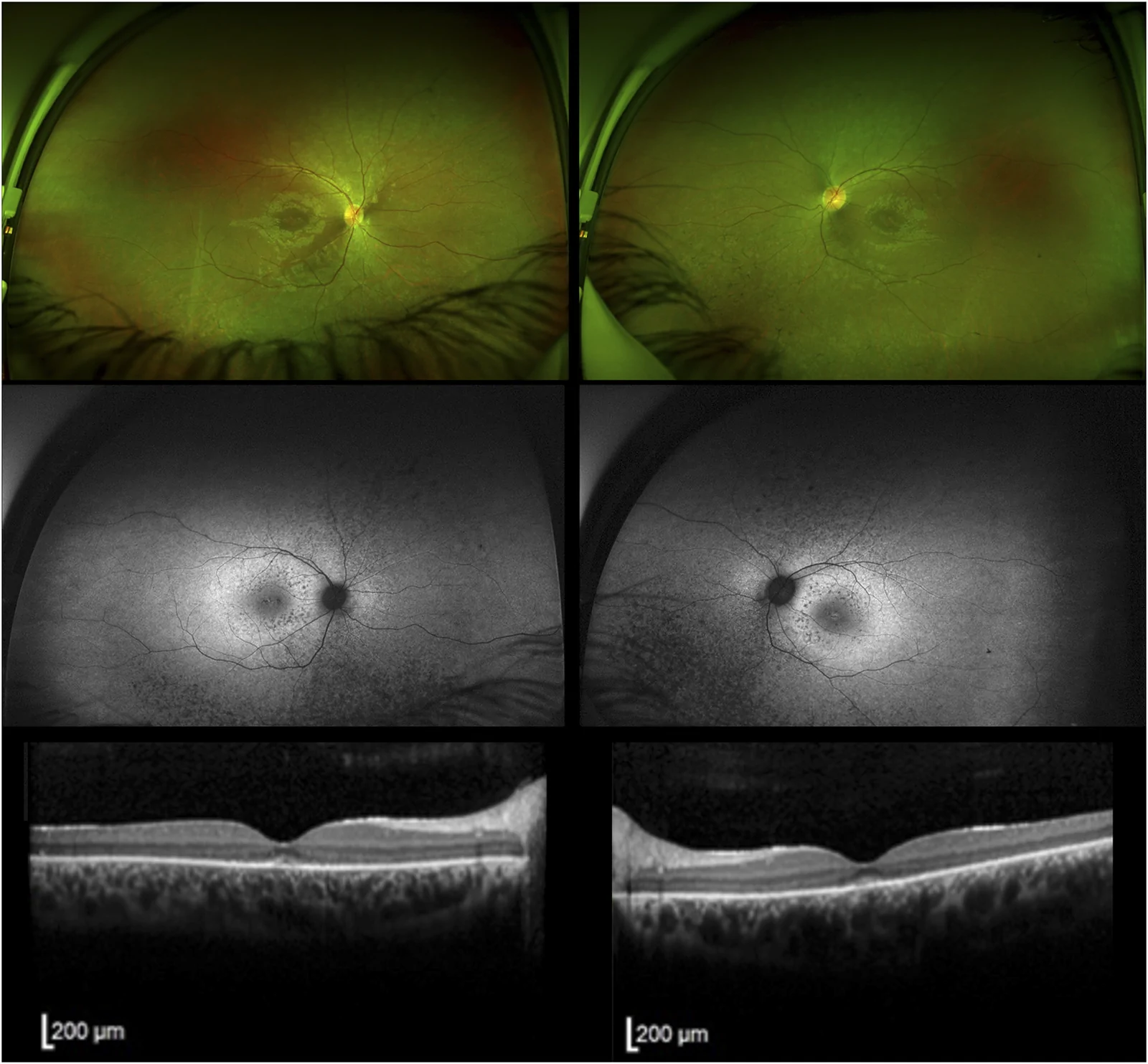
Fundus features of patient 11.1: Preserved optic disc, attenuated vessels, mottled RPE aspect in posterior pole, nasal inferior mid-periphery, and atrophic macula. OCT scan shows foveal-sparing OU.

### Molecular genetics and genotype-phenotype correlation

Twenty-four different variants described in the *CERKL* gene were identified (Figure 2). The c.847C>T nonsense variant in exon 6 was the most common variant in this cohort, identified in 24 homozygous out of 50 families and in 10 compound heterozygous. The second most common variant was c.237_238+13del, identified in 2 homozygous and in 1 compound heterozygous family. Among the 24 variants identified in our cohort, 3 were missense, 2 were nonsense, 4 were frameshift, 14 were splice site alteration, and 1 was a large deletion. Fifteen were classified as pathogenic, 3 as likely pathogenic, and 5 as VUS. Four patients were heterozygous with one VUS, and one patient was homozygous for a VUS. All four heterozygous patients had a phenotype for *CERKL* retinopathy and therefore were included in the cohort. Two variants classified as VUS were not previously described in Clinvar records, so are candidates’ novel alleles: exon3 deletion and c.898+5G>A. Patient 45.1 homozygous for a VUS in *CERKL* exhibited a phenotype consistent with *CERKL* retinopathy but was excluded from the analysis as it was not considered a confirmed null variant.

Patients’ variants and their molecular characteristics are summarized in Table 2. Thirty-five patients were homozygous.

**TABLE 2:** Data of variants in the cohort.

Nucleotide change	Location	Protein change	Variant type	ACMG classification	Gnomad v4.1.0(%)	First report
c.38dup	Exon 1	p.Glu14Glyfs*47	Frameshift indel	Pathogenic	no frequency	PMID 14681825, 24043777, 23591405
c.182T>A	exon1	p.Val61Glu	Missense	Likely pathogenic	0,003	PMID 30902645
c.222del	exon1	p.Gln74Hisfs*28	Frameshift	Pathogenic	0,001	PMID 14681825
c.238+1G>A	intron1	p.?	Splice donor	Pathogenic	0,00006	PMID 18055789
c.237_238 + 13del	Intron 1	p.Gly80fs	Insertion and deletion	Pathogenic	0,0006	PMID 14681825
c.239-1G>A	intron1	p.?	Splice acceptor	Pathogenic	0,0008	na.
c.356G>A	exon2	p.Gly119Asp	Missense	Likely pathogenic/Pathogenic	0,006	PMID 26766544
c.481+2T>G	Intron 2	p.?	Splice donor	Pathogenic	no frequency	PMID 14681825
deletion (exon3)	Exon 3	p. ?	Frameshift	VUS	no frequency	This study
c.677+5G>T	Intron 4	p.?	Splice donor	VUS	0,0007	PMID 17576681, 9536098, 28492532
c.756-1G>C	Intron 5	p.?	Splice acceptor	Pathogenic	no frequency	PMID 14681825
c.847C>T	Exon 6	p.Arg283*	Nonsense	Pathogenic	0.05	PMID:14681825
c.895+1G>A	Intron 6	p.?	Splice donor	Pathogenic	0,0009	PMID 14681825
c.898+2C>G	Intron 6	p.?	Splice donor	Pathogenic	0,003	PMID 14681825
c.898+5G>A	Intron 6	p.?	Splice donor	VUS	no frequency	This study
c.1073 + 3_1073+6delAAGT	closest exon 8	p.?	Splice donor	Likely pathogenic	0,0003	PMID 27874104, 28041643
c.1152-1G>C	Intron 8	p.?	Splice acceptor	Pathogenic	no frequency	PMID 14681825, 24043777, 23591405
c.1160-10T>G	Intron 10	p.?	Splice acceptor	Likely pathogenic	0.04	na.
c.1237+5G>A	Intron 10	p.?	Splice donor	VUS	no frequency	na.
c.1238-10T>G	Intron 10	p.?	Splice acceptor	VUS	0.04	na
c.1305C>A	Exon 11	p.Cys435*	Nonsense	Pathogenic	0,007	PMID 14681825,24043777, 23591405
c.1392_1395dup	Exon 12	p.Pro466Serfs*24	Frameshift	Pathogenic	0,006	na.
c.1470dup	exon13	p.Thr491Hisfs*3	Frameshift	Pathogenic	no frequency	PMID 14681825, 24043777
c.1576G>A	Exon 13	p.Asp526Asn	Missense	VUS	na.	na.

### Genotype-phenotype correlation analysis

Individuals homozygous with nonsense variant c.847C>T presented as CORD (19/24), with early symptoms evident from 7 years of age (proband 22.1).

Two Homozygous c.237_238+13del individuals (8.1 and 12.1) presented with the CORD phenotype, with initial symptoms of reduced central visual acuity at 17 and 21 years of age, respectively. Fundus examination of those patients showed relatively preserved optic disc and vessels, peripapillary atrophy, no pigments in the posterior pole, and atrophic macula.

Macular dystrophy phenotype was found in two individuals with compound heterozygous variants (Patient 24.1 and 31.1). The next paragraph presents a detailed description of these two cases who did not have conclusive molecular diagnosis. This discussion is provided as hypothesis-generating observations**.**

Patient 24.1 is a 37-year-old male proband who noticed a decrease in his central vision at the age of 12. Bilateral central atrophy was evident on fundoscopy and imaging. AF imaging showed central loss of signal consistent with atrophy, with a well demarcated margin of hyperautofluorescence around the atrophic area and extending to the disc. He is compound heterozygous for a VUS missense c.1576G>A, p.Asp526Asn and a pathogenic c.237_238+13del, p.Gly80fs variants for *CERKL*. Only one pathogenic variant was found, so further functional studies are necessary to conclude the molecular diagnosis for this case.

Patient 31.1 is a female patient who has experienced photophobia and a progressive decrease in her visual acuity since 12 years of age. She has a 26-year history of disease progression. No consanguinity was referred. Visual acuity was 20/400 OU and fundus phenotype was consistent with macular dystrophy with well demarcated macular atrophy OU. The genotype result was a compound heterozygous c.1238-10T>G pathogenic variant and an exon 3 deletion VUS not yet reported in the Clinvar database. The exon 3 deletion is a gross genomic loss encompassing exon 3 of the *CERKL* and is predicted to be in-frame, preserving the integrity of the reading frame. This variant has not before been reported in the literature in individuals affected with *CERKL*-related conditions. Experimental studies and prediction algorithms are not available or were not evaluated, and the functional significance of this variant is currently unknown. Further functional studies are necessary to conclude the molecular diagnosis for this patient.

Patient 45.1 is a female proband who reports poor visual acuity and photophobia since age 15 and has been followed for 30 years. Present visual acuity is light perception in OD and NLP in OS. The homozygous c.898+5G>A variant was identified in an intronic region of *CERKL* gene and no information regarding splicing site impact is available. This variant meets support (PM2) ACMG criteria, since the allele is absent in gnomAD, 1000Genomes, and ABraOM databases and support (PM3) ACMG criteria, as found as a homozygous variant. In silico prediction algorithms disagree that this variant has any protein impact. This variant has not previously been reported in Clinvar literature; accordingly, it is classified as a VUS. Therefore, this patient’s genetics findings were considered inconclusive for *CERKL*-related IRD retinopathy.

## Discussion

*CERKL* is an ultrarare gene implicated in IRD in different ethnic populations [7, 21–24]. In this Brazilian cohort, the *CERKL* gene accounts for 2% of the total positive tests for IRD, which is consistent with the 2.5% from a previous publication from our group [25] in 2018.

The relative frequency rates in diverse IRD in Brazil are comparable and aligned with the 0.72% reported in the global pool from the Foundation Fighting Blindness Clinical Consortium [26], which corroborates that *CERKL* is an uncommon contributor to IRD.

Linear regression analysis was performed to evaluate the association between disease duration and visual acuity in *CERKL*-related retinal dystrophy patients, stratified by genotypic severity into double-null (DN) — carrying two loss-of-function alleles — and non-double-null (NDN) groups.

The analysis indicates that complete biallelic loss of *CERKL* function is associated with a more rapid and statistically predictable decline in visual acuity compared to the group retaining residual protein function.

The most frequent variant found was the c.847 C>T nonsense variant, with an allele frequency of 55.67%. This variant, c.847C>T, was the first described in Spanish families by Bayes e Tuson and is the most frequent found in the literature [24]. The allele frequency was reported to be 88.9% in Spain and 76.5% in Portugal [27]. The relative high frequency of the c.847C>T variant in our cohort may be explained by historical records suggesting that immigration to Brazil predominantly originated from Portugal and Italy [28], confirmed by a publication regarding genetic ancestry in Brazilian population [29].

Patients with this homozygous and compound heterozygous variant presented clinically as cone rod dystrophy phenotype. Compound heterozygous variants also included two patients with macular dystrophy phenotype (patients 24.1 and 31.1), an uncommon description for *CERKL*-related retinopathy.

*CERKL*-related retinopathy can present as early as 7 years old with a median of 18 years, with decrease in central vision associated with nyctalopia symptoms, and an early drop in visual acuity. Clinically, an optic disc not severely impaired, peripapillary atrophy, sparse mottled pigmentation, and central atrophy were observed. Sparse mottled RPE abnormalities were observed in the CORD phenotype with more diffuse and dense pigmentation abnormalities in advanced cases. These evolutive presentations have been described by other authors for *CERKL*-related retinopathy [23, 27]. Early macular involvement as a key feature and concomitant involvement of both rods and cones in full-field ERG points to a CORD phenotype presentation documented in other publications [8, 12] for 847C>T and c.238+1G>A variants. Avila et al. described how most of the patients in the Finland cohort had juvenile unspecified macular dystrophy as their initial diagnosis, while two patients had Stargardt disease as the initial diagnosis, due to Stargardt-like phenotype. Studies in murine retina suggest *CERKL* is more highly expressed in cones compared to rods [14, 30]. This information also strengths our hypothesis that *CERKL* related-retinopathy manifest predominantly as CORD phenotype.

The macular phenotype was noted in 2 individuals in our cohort (patients 24.1 and 31.1), both of whom had heterozygous compound variants but lacked a conclusive molecular diagnosis.

Patient 24.1 is compound heterozygous for c.1576G>A, pAsp526Asn and c.237_238+13del, p.Gly80fs variants for *CERKL* with bilateral hyperautofluorescent. This finding was also described by two authors for *CERKL* compound heterozygous cases with c.375C>G and c.193G>T variants (Avila et al) and c.1393C>T and c.316C>A variants [31].

Patient 31.1 is a female proband who was compound heterozygous for c.1238-10T>G and deletion exon3 variant for *CERKL*. The exon 3 deletion variant was not previously reported in the Clinvar database. This variant disrupts a region corresponding to the N-terminal portion of the diacylglycerol catalytic domain, which is responsible for the protein integrity of kinase fold. This evidence could disestablish a clinically significant region of the protein [14, 17] and means it is likely to be disease-causing. This may represent a new variant; additional functional studies are required to confirm its status.

Electrophysiological assessment was scarce, with only 3/52 individuals having performed the exam. As no clear correlation between ERG findings and clinical data exists [24], retinal electrical activity profile analysis of all individuals of the cohort would clarify the comprehension of *CERKL*- related retinopathy phenotype. The most consistent hallmark is a concomitant and parallel progressive impairment of both rod and cone systems [8, 23, 27].

Twenty-four different variants were found in our cohort. The Brazilian population is highly diverse, probably because of multiple admixture events between Indigenous American, African, and European parental sources; this fact could explain why there were a significant number of different variants in this cohort. A recent publication from the DNABR project, an initiative of Brazilian government to map the genetic diversity of the Brazilian population, has sequenced thousands of Brazilian genomes and identified more than 8 million previously unknown single nucleotide variants (SNVs) not present in the main public datasets.

Regarding gene function, strong evidence was provided for mitochondrial localization of the CERKL protein in the mammalian retina, specifically the isoforms containing exon 5 [32]. Depletion of CERKL levels in knockdown/knockout mouse retinas were shown to increase autophagy, mitochondrial fragmentation, alteration of mitochondrial distribution, and dysfunction of mitochondrial-dependent bioenergetics and metabolism, issues related to mitochondrial metabolic pathways. These results suggest that the *CERKL* gene acts as a regulator of mitochondrial biology and metabolism with a retinal resilience role.

The phenotype of *CERKL*-related retinopathy is predominantly CORD with an early decrease in visual acuity. The macular dystrophy phenotype was found in 2 patients, both compound heterozygous for *CERKL* variants, and provided hypothesis-generating observations that could not provide a conclusive molecular diagnosis. In addition to OMIM classifying this gene as an RP IRD, we believe *CERKL* has CORD phenotype, in concordance with evidence described in the literature.

### Study limitations

The primary limitations of this study include its retrospective design and the incomplete availability of imaging data for all patients. Furthermore, the absence of segregation analysis for several families precludes the definitive confirmation of the genetic etiology in certain cases. This was particularly relevant for the presumed compound heterozygous *CERKL*-related cases, where segregation would have provided essential evidence for the molecular interpretation and pathogenicity of the identified variants.

In addition to these constraints, the use of various testing platforms across different laboratories also introduced a degree of methodological heterogeneity. Detailed information regarding test types is listed in Supplementary Table S1. While targeted NGS panels and whole-exome sequencing (WES) provide robust detection for single-nucleotide variants and small insertions/deletions (indels), their sensitivity for structural variants can be inconsistent. Since not all platforms utilized specialized copy number variant (CNV) calling algorithms, it is possible that large deletions were under-detected in patients who underwent basic panel sequencing. This limitation underscores the necessity of standardized diagnostic pipelines in multicenter cohorts to ensure uniform variant detection rates.

## Data Availability

The original contributions presented in the study are included in the article and Supplementary Material, further inquiries can be directed to the corresponding author.
