# Supplementary material for: CERKL-related inherited retinal dystrophy in a Brazilian cohort: genotype-phenotype correlation
Source: Exp Biol Med (Maywood). 2026 Sep 23;251:10935. doi: 10.3389/ebm.2026.10935 (PMC13642226; doi:10.3389/ebm.2026.10935)
Supplement: Supplementary file 1 [file Table1.pdf]

Supplementary Table 1: Test type, platform, laboratory provider, year and country

#Patient	Test type	CNV analysis included? (Yes/No/Unknown)	Platform	Laboratory/Provider	Year	Country
**1,1**	NGS panel	Yes	Illumina	Mendelics	2017	Brazil
**2,1**	NGS panel	Yes	Illumina	Invitae	2018	Brazil
**2,2**	NGS panel	Yes	Illumina	Invitae	2018	Brazil
**3,1**	NGS panel	Yes	Illumina	Mendelics	2018	Brazil
**4,1**	NGS panel	Yes	Illumina	Invitae	2021	Brazil
**5,1**	NGS panel	Yes	Illumina	Mendelics	2018	Brazil
**6,1**	NGS panel	Yes	Illumina	Mendelics	2017	Brazil
**7,1**	NGS panel	Yes	Illumina	Invitae	2017	Brazil
**8,1**	NGS panel	Yes	Illumina	Mendelics	2020	Brazil
**9,1**	NGS panel	Yes	Illumina	Invitae	2021	Brazil
**10,1**	NGS panel	Yes	Illumina	Molecular Vision	2011	USA
**11,1**	NGS panel	Yes	Illumina	Mendelics	2017	Brazil
**12,1**	NGS panel	Yes	Illumina	Mendelics	2021	Brazil
**13,1**	NGS panel	Yes	Illumina	Mendelics	2017	Brazil
**14,1**	NGS panel	Yes	Illumina	Molecular Vision	2019	Brazil
**15,1**	NGS panel	Yes	Illumina	Invitae	2021	Brazil
**16,1**	NGS panel	Yes	Illumina	Mendelics	2021	Brazil
**17,1**	NGS panel	Yes	Illumina	Genoma Humano	2020	Brazil
**18,1**	NGS panel	Yes	Illumina	Asper	2010	Estonia
**19,1**	NGS panel	Yes	Illumina	Mendelics	2022	Brazil
**20,1**	NGS panel	Yes	Illumina	Geneone	2021	Brazil
**21,1**	NGS panel	Yes	Illumina	Invitae	2021	Brazil
**22,1**	NGS panel	Yes	Illumina	Mendelics	2017	Brazil
**23,1**	NGS panel	Yes	Illumina	Mendelics	2021	Brazil
**24,1**	NGS panel	Yes	Illumina	NIM Genetics	2018	Brazil
**25,1**	NGS panel	Yes	Illumina	Dasa	2023	Brazil
**26,1**	NGS panel	Yes	Illumina	Dasa	2024	Brazil

Supplementary Table 1: Test type, platform, laboratory provider, year and country (cont.)

#Patient	Test type	CNV analysis included? (Yes/No/Unknown)	Platform	Laboratory/Provider	Year	Country
**27,1**	NGS panel	Yes	Illumina	Invitae	2021	Brazil
**28,1**	NGS panel	Yes	Illumina	Invitae	2021	Brazil
**29,1**	NGS panel	Yes	Illumina	Geneone	2023	Brazil
**30,1**	NGS panel	Yes	Illumina	Invitae	2021	Brazil
**31,1**	NGS panel	Yes	Illumina	Invitae	2021	Brazil
**32,1**	NGS panel	Yes	Illumina	Geneone	2022	Brazil
**33,1**	Exoma	Yes	Illumina	Genética Médica e Forense	2023	Brazil
**34,1**	NGS panel	Yes	Illumina	Mendelics	2023	Brazil
**35,1**	NGS panel	Yes	Illumina	Invitae	2021	Brazil
**35,2**	NGS panel	Yes	Illumina	Invitae	2021	Brazil
**36,1**	Exoma	Yes	Illumina	Mendelics	2022	Brazil
**37,1**	NGS panel	Yes	Illumina	Invitae	2022	Brazil
**38,1**	NGS panel	Yes	Illumina	Invitae	2021	Brazil
**39,1**	NGS panel	Yes	Illumina	Invitae	2021	Brazil
**39,2**	NGS panel	Yes	Illumina	Invitae	2021	Brazil
**40,1**	NGS panel	Yes	Illumina	Invitae	2021	Brazil
**41,1**	NGS panel	Yes	Illumina	Pasteur	2025	Brazil
**42,1**	NGS panel	Yes	Illumina	Invitae	2022	Brazil
**43,1**	NGS panel	Yes	Illumina	Invitae	2022	Brazil
**44,1**	NGS panel	Yes	Illumina	Invitae	2022	Brazil
**45,1***	NGS panel	Yes	Illumina	Geneone	2022	Brazil
**46,1**	NGS panel	Yes	Illumina	Invitae	2022	Brazil
**47,1**	NGS panel	Yes	Illumina	Invitae	2022	Brazil
**48,1**	NGS panel	Yes	Illumina	Blueprint Genetics	2022	Ireland
**49,1**	NGS panel	Yes	Illumina	Dasa	2023	Brazil
**50,1**	NGS panel	Yes	Illumina	Invitae	2022	Brazil

*Patient 45.1 is a homozygous vus and was excluded from the analysis
